# Molecular epidemiology and comparative genomics of *Campylobacter concisus* strains from saliva, faeces and gut mucosal biopsies in inflammatory bowel disease

**DOI:** 10.1038/s41598-018-20135-4

**Published:** 2018-01-30

**Authors:** Karina Frahm Kirk, Guillaume Méric, Hans Linde Nielsen, Ben Pascoe, Samuel K. Sheppard, Ole Thorlacius-Ussing, Henrik Nielsen

**Affiliations:** 1 Department of Infectious Diseases, Aalborg University Hospital, Aalborg, Denmark; 2 Department of Clinical Medicine, Aalborg University, Aalborg, Denmark; 3 The Milner Center for Evolution, Department of Biology and Biochemistry, Bath University, Bath, United Kingdom; 4 Department of Clinical Microbiology, Aalborg University Hospital, Aalborg, Denmark; 5 Department of Zoology, University of Oxford, Oxford, UK; 6 Department of Gastrointestinal Surgery, Aalborg University Hospital, Aalborg, Denmark

## Abstract

*Campylobacter concisus* is an emerging pathogen associated with inflammatory bowel disease (IBD), yet little is known about the genetic diversity of *C. concisus* in relation to host niches and disease. We isolated 104 *C. concisus* isolates from saliva, mucosal biopsies and faecal samples from 41 individuals (26 IBD, 3 Gastroenteritis (GE), 12 Healthy controls (HC)). Whole genomes were sequenced and the dataset pan-genome examined, and genomic information was used for typing using multi-locus-sequence typing (MLST). *C. concisus* isolates clustered into two main groups/genomospecies (GS) with 71 distinct sequence types (STs) represented. Sampling site (p < 0.001), rather than disease phenotype (p = 1.00) was associated with particular GS. We identified 97 candidate genes associated with increase or decrease in prevalence during the anatomical descent from the oral cavity to mucosal biopsies to faeces. Genes related to cell wall/membrane biogenesis were more common in oral isolates, whereas genes involved in cell transport, metabolism and secretory pathways were more prevalent in enteric isolates. Furthermore, there was no correlation between individual genetic diversity and clinical phenotype. This study confirms the genetic heterogeneity of *C. concisus* and provides evidence that genomic variation is related to the source of isolation, but not clinical phenotype.

## Introduction

*Campylobacter concisus* is an emerging pathogen that is a part of the commensal human oral microbiota^1^. Recently, the species has been associated with diseases of the gastrointestinal tract, such as Barrett’s esophagus^2^, prolonged diarrhoea^3,4^ and inflammatory bowel disease (IBD)^5–8^. Diversity within *C. concisus* populations may explain differences in pathogenic activity as well as detection of isolates in both patients and healthy control individuals^9,10^. However, the extent of genetic diversity of isolates from different disorders has not been well described, and the diversity of multiple isolates from the same individual is unknown.

Various typing methods such as amplified fragment length polymorphisms (AFLP)^11,12^, 23 S rRNA PCR^12,13^ and multi-locus sequence typing MLST^14–16^ have previously been used for strain typing of *C. concisus*. MLST is widely used as a method for typing that can identify lineages and population structures in a microorganism^17^, and the method has been shown to have a high discriminatory power for *Campylobacter jejuni* and *Campylobacter coli*^18^ and for emerging *Campylobacter* species^19^. One of the advantages of MLST is that sequence data can be easily exchanged between laboratories for use in global epidemiological research.

In general, these studies have shown a consistent division of *C. concisus* isolates into two main clusters or genomospecies (GS), regardless of typing method. A correlation with clinical presentation has been suggested, since isolates from diarrheic individuals were overrepresented in the same GS in previous studies^12,13^. However, this subdivision was not found in subsequent studies from oral isolates^15^, or diarrheic faecal samples^16^ where pathogenic isolates were equally present in both GS^16^. Phylogenetic differentiation has also been reported among isolates from gastroenteritis and Crohn’s disease, implying genetic differences associated with diseasephenotypes^9^. These studies provide a good basis for considering the molecular epidemiology of *C. consisus* but further work, with large well characterised isolate collections, is necessary to understand how population structure relates to clinical significance in these highly diverse, recombining bacteria^15^. Moreover, most studies have been performed with isolates from saliva and faeces, whereas limited information from mucosal biopsy isolates is available. Only few studies have used whole genome sequencing to compare *C. concisus* isolates^9,20,21^. It has been proposed that exotoxin 9, used as a proxy for a group of conserved genes on the UNSWCD plasmid, may be involved in intracellular survival, since this was only detected in the highly invasive strains^22^. Another gene with a potential role in *C. concisus* pathogenicity is *zot*, encoding the Zot toxin that targets intercellular tight junctions^23–25^. Recently, Chung *et al*. analysed the genomes of 27 *C. concisus* isolates, mostly from the oral cavity^20^. In that study, novel genomic islands containing type IV secretion systems, putative effector proteins and CRISPR-associated proteins were identified, with different prevalence between genomospecies.

In this study we investigated oral, gut mucosal and faecal isolates sampled from patients with inflammatory bowel disease (IBD), diarrhoae/gastroenteritis (GE), and healthy controls (HC). We used MLST, whole genome sequencing, core- and accessory genome characterization to investigate the diversity of a large number of isolates from different anatomical sites within individuals, including gut mucosal biopsies from healthy controls.

## Results

### Population structure and epidemiology

**Figure 1:**
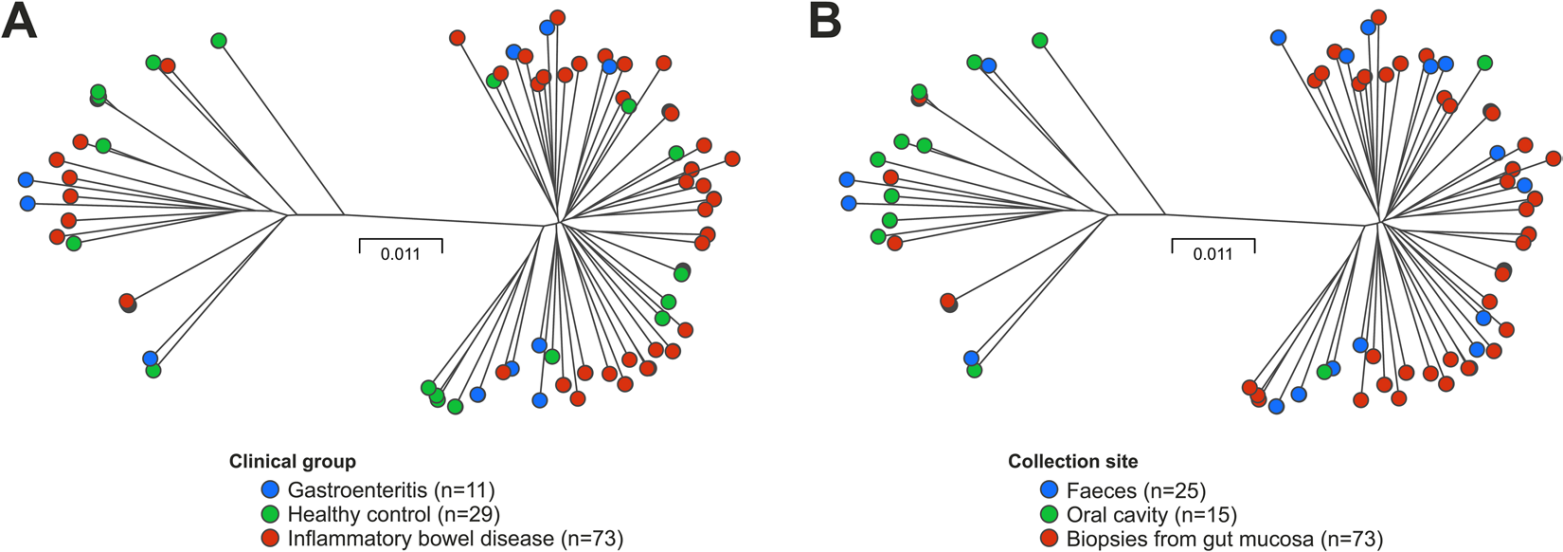
Genetic relatedness of *C. concisus* isolates. Each dot represents a single isolate, coloured according to: (**A**) disease phenotype (red = IBD, blue = gastroenteritis (GE), green = healthy controls (HC)) and (**B**) sample collection site (red = gut mucosal biopsies, blue = faeces, green = saliva). Left and right clusters represent genomospecies (GS) I and II, respectively. The proportion of isolates from IBD patients and healthy controls in the two genomospecies was not statistically different (p = 1.0), whereas isolates from saliva were more frequent in GS I compared to gut mucosal biopsy isolates, more frequent in GS II (p < 0.001). Phylogenetic trees were created from concatenated sequences of seven housekeeping genes.

In accordance with previous findings, *C. concisus* isolates clustered into two main groups/genomospecies (GS). When annotated according to disease status, there was no difference between GS, as isolates deriving from IBD patients, diarrheic patients and healthy controls were present in both clusters (p = 1.00) (Fig. 1A). However, when assessing anatomical site of collection, GS II isolates predominated in gut mucosal samples and GS I in oral samples (p < 0.0001) (Fig. 1B). Faecal isolates were equally distributed in both clusters, independent of disease status (p = 1.00). MLST using the combination of loci defined by Miller *et al*.^19^ revealed a high diversity of *C. concisus* with 71 ST’s and the following number of alleles: *aspA:*63, *atpA:*65, *glnA:*62, *gltA:*64, *glyA:*62*, ilvD:*64 and *pgm:*63. For comparison, typing by 16 S rRNA, 23 S rRNA sequences and MLST using other previously described loci^15^ was performed. These typing methods showed consistent results with isolates clustering into two groups (data not shown).

Two or more isolates were collected from 27/41 patients (18 IBD, 2 GE, 7 HC). The mean number of isolates collected per individual was 3 (1–12). Isolates from 17/27 patients (63%) were genetically different, with isolates from seven individuals (4 IBD, 1 GE, 2 HC) being represented in both GS. These findings were independent of clinical presentation and sampling site (Supplementary Figure 1).

### Pangenome content analysis and identification of genes involved in colonisation

**Figure 2:**
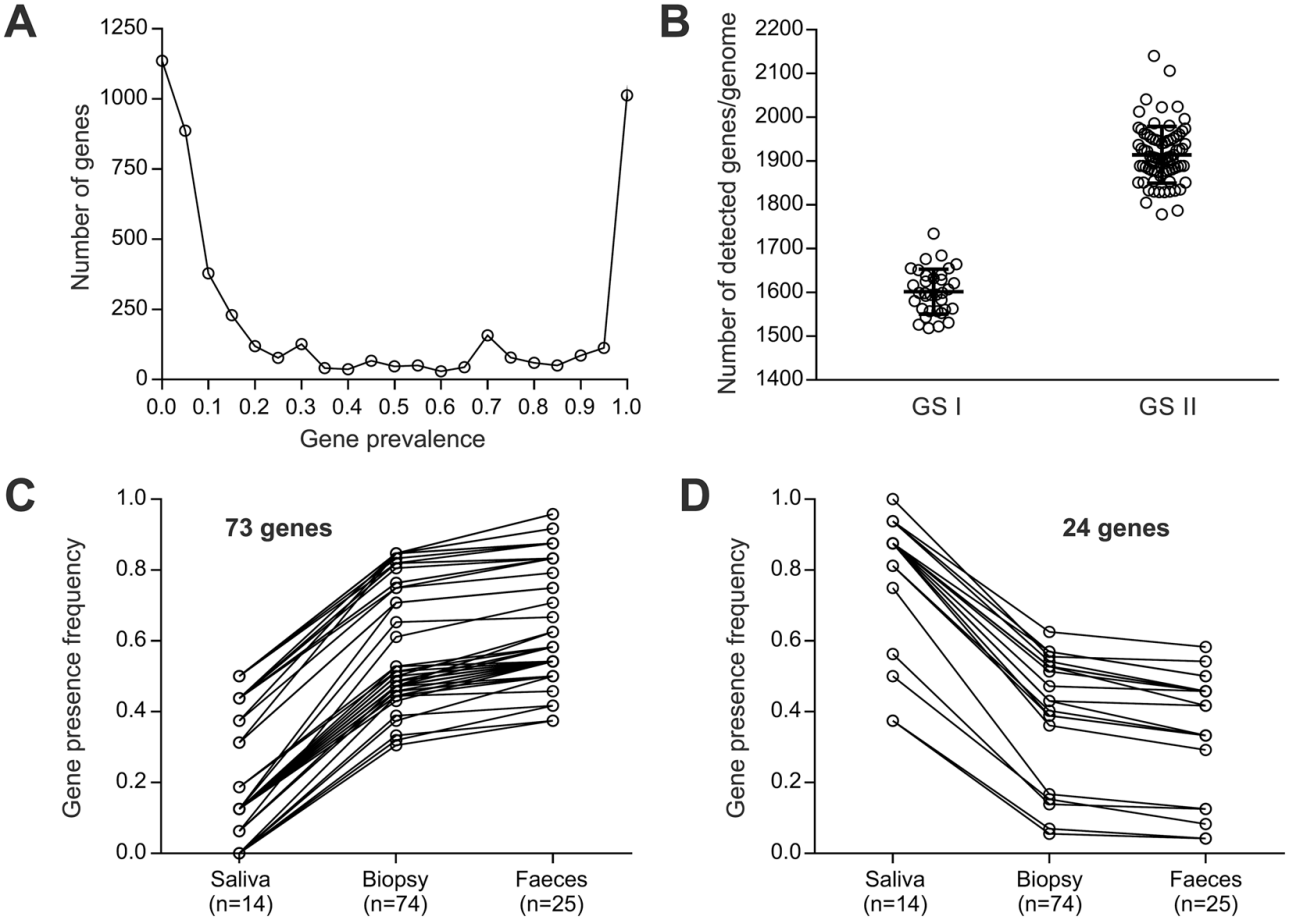
Panel A: Overview of the pangenome and prevalence of detected genes per genome. A total of 864 core genes were present in all isolates and 1,095 genes were present in > 95% isolates. Panel B: Isolates belonging to GS II had a higher number of detected genes per genome compared to isolates from GS I. In average there were 313 ± 13 more genes per genomes in GS II isolates (1,914 ± 7, n = 78) compared to GS I isolates (1602 ± 9, n = 34) (p < 0.0001). Panel C: Seventy-three genes were found to increase in prevalence from oral isolates to gut mucosal isolates and to faecal isolates, with a minimum increase of 30% from saliva to gut mucosal biopsies. Panel D: Twenty-four genes decreased in prevalence from saliva to gut mucosal biopsies and to faeces, with a minimum 30% decrease from saliva to gut mucosal biopsies.

We examined the prevalence and variation of the 4,798 genes from the pangenome of 113 *C. concisus* genomes. A total of 864 core genes were present in all isolates and 1,095 genes were present in >95% isolates (Fig. 2A). The number of detected genes per genome differed between the two GS. There was an average of 313 ± 13 more genes per genomes in GS II isolates (1,914 ± 7, n = 78) compared to GS I isolates (1602 ± 9, n = 34) (Fig. 2B), which was a significant difference (Unpaired two-tailed *t* test; *t* = 24.93, df = 110; p < 0.0001). Consequently, the GS-specific core genome size was also higher in GS II (1,367 genes, or 71.4% of the GS II average genome size) than in GS I (975 genes shared by all GS I isolates, or 60.9% of the GS I average genome size), with a slight overrepresentation of GS II core genes predicted to be involved in amino-acid and carbohydrate metabolism (Supplementary Table 1). Also, there were more than twice as many genes absent from all GS I isolates and present in at least one GS II isolate than the opposite (1,537 vs. 667 genes, respectively). Overall, this suggests extensive genomic divergence between GS I and GS II lineages in *C. concisus* that could be related to functional variation.

We identified genes that varied in prevalence in different groups of isolation sites. *C. concisus* was isolated from 12 distinct body sites. Of these, ten were from biopsies of the gastrointestinal tract (proximal/distal ileal-anal-pouch, ileum, terminal ileum, cecum, ascending colon, transverse colon, descending colon, sigmoideum and rectum). In total, our dataset comprised of 14 isolates from saliva, 74 from gastrointestinal biopsies, and 25 from faeces (including the nine previously published genomes). We identified 73 genes increasing in prevalence from saliva to biopsies to faeces, that had a minimum 30% increase in prevalence between saliva and biopsies (Fig. 2C, Supplementary Table 2). Additionally, we identified 24 genes decreasing in prevalence from saliva to biopsies to faeces, and that had a minimum 30% decrease in prevalence between saliva and biopsies (Fig. 2D, SupplementaryTable 2). Of these 97 genes, 60 had a COG functional assignation, the rest being composed of predicted hypothetical proteins (Supplementary Table 2). In isolates from gut mucosal biopsies and faeces, there was an over-representation of functions involved in amino acid-, carbohydrate- and lipid transport and metabolism compared to isolates from the oral cavity. In contrast, oral isolates had more genes involved in cell wall/membrane biogenesis and inorganic ion transport, compared to enteric strains (Supplementary Table 2).

### Exotoxin 9 and *zot*

In the 104 isolates from this study, 67 (64%) had either *zot* or exotoxin 9 DNA, or both. Eight isolates had *zot* only, 50 exotoxin 9 only, and nine had both *zot* and exotoxin 9. In total, 59 (57%) isolates from 26 different patients (IBD n = 15, HC n = 9, GE n = 2), had exotoxin 9 DNA, with the majority being gut mucosal isolates (n = 42) (Tables 1 and 2). There was noticeably fewer isolates with exotoxin 9 only from IBD patients (37%) compared to HC (70%) and GE patients (71%). Nine isolates were positive for both *zot* and exotoxin 9 DNA, and all these nine isolates were from IBD patients with the majority (n = 6) originating from gut mucosal isolates. Isolates positive for *zot* only, were more prevalent in GS I (6/18) compared to GS II (2/39) (p = 0.039), whereas isolates positive for exotoxin 9 only, was higher in GS II (20/39) compared to GS I (4/18) (p = 0.004). Isolates with both putative virulence factors were not significantly different between the two genomospecies (p = 0.56).

**Table 1:** Isolates positive for *zot* and/or exotoxin 9 in samples according to clinical presentation (n = 104).

Gene	IBD (n = 66)	HC (n = 31)	GE (n = 7)
*zot* only	3 (4.5%)	4 (12.9%)	1 (14.3%)
Exotoxin 9 only	26 (39.3%)	19 (61.2%)	5 (71.4%)
*zot* + exotoxin 9	9 (13.6%)	0	0

**Table 2:** Isolates positive for *zot* and/or exotoxin 9 in samples according to sample collection site (n = 104).

Gene	Saliva (n = 13)	Biopsy (n = 71)	Stool (n = 20)
*zot* only	2 (15.3%)	3 (4.2%)	3 (15%)
Exotoxin 9 only	4 (30.7%)	36 (50.4%)	10 (50%)
*zot* + exotoxin 9	1 (7.1%)	6 (8.4%)	2 (10%)

**Figure 3:**
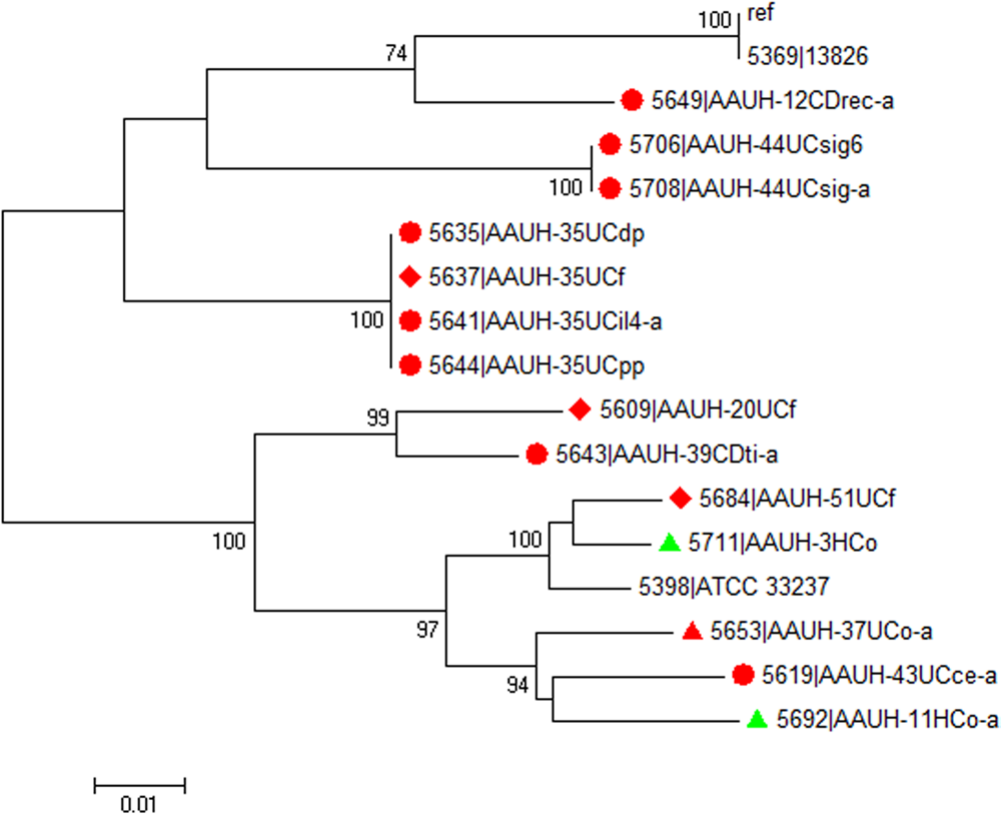
A phylogenetic tree based on the concatenated sequences of the *zot* gene, in 14 *C. concisus* isolates. Colours indicate clinical presentation (red = IBD, green = HC), shapes indicate sampling site: (dots = gut mucosal biopsies, triangles = saliva samples, diamonds = faecal samples).

The *zot* gene has previously been described in the reference isolate *C. concisus* 13826 (ATCC BAA-1457) and was also detected in ATCC 33237. These sequences were included in a phylogenetic analysis, which showed grouping into two main clusters (Fig. 3). Three isolates were not included in the phylogenetic analysis because the genes were located at the end of a contig and were therefore incomplete (AAUH-2010376221 (faecal isolate, diarrheic patient, bp = 670), 14HC (mucosal isolate, healthy control, bp = 826), and 11HC (faecal isolate, healthy control, bp = 702)). They were, however, included for evaluation of polymorphisms where possible. No patient had more than one type of *zot*, even when detected in samples from different sites. When analyzing nucleotide sequence data and amino acid composition, we found that none of our isolates had the *zot*^350–351AC^ polymorphism previously described by Mahendran *et al*.^24^. The *zot*^multiple^ was detected in three isolates (13826, 44UCsig6 and 44UCsig-a). We found the *zot*^808T^ polymorphism only in one mucosal isolate, interestingly from a healthy control (14HC). The amino acid substitutions from the polymorphisms sites were equivalent to those previously reported by Mahendran *et al*.^24^, with a substitution of valine at position 270. We did not find any other nucleotide polymorphisms or amino acid substitutions in our data set that correlated to clinical presentation.

### Other putative virulence genes

In our isolates, we identified some of the CRISPR-associated genes and plasmid integration island genes, previously described by Chung *et al*.^20^. Similar to their findings, the prevalence of some Cas-proteins were specific to genomospecies, such as Cas1_1, Cas2 and Cas3 which were only found in GSII (p < 0.001). In general, CRISPR-associated genes and plasmid integration island genes were generally more common in isolates from genomospecies II, but there was no difference between clinical presentation (Supplementary Table 3).

## Discussion

We present analyses of 104 *C. concisus* isolates from both IBD patients and healthy controls, including samples collected from different anatomical sites in the same individual. Previous studies have shown that *C. concisus* is genetically heterogeneous, and we now document that this diversity exists throughout the entire gastrointestinal tract, regardless of clinical presentation. Reports on the molecular epidemiology of *C. concisus* have primarily included isolates from saliva and faeces with a limited number of isolates from gut mucosa^9,15,20^. We now expand considerably the examination of *C. concisus* isolates by culture from gut mucosal biopsies from multiple sites of the intestine. An important finding is that for several genes the relative frequency increases or decreases in isolates sampled along the gastrointestinal tract. It is possible that as the host niche and microbiome varies, different genotypes acquire a competitive advantage and this may be related to the pathogenicity of *C. concisus* at the gut epithelium. It has been suggested that the oral cavity may be the natural reservoir for *C. concisus* colonisation, and genetically different isolates have been isolated from the oral cavity of the same individual^1^. We found that genetic differences exist between isolates sampled at multiple anatomical sites and that there can be different genotypes in the same clinical sample. This suggests that multiple different isolates can potentially colonize the gut mucosa.

Some of the isolates collected in this study were sampled at different time points, for one patient four years apart. The current understanding of transmission and duration of *C. concisus* colonisation in humans is very limited. While colonisation of the human oral cavity may facilitate human to human transmission, *C. concisus* has also been isolated from domestic pets^26^, as well as from chicken and beef samples^27^. Comparing the genetic diversity between isolates recovered from different mammalian species has not been performed, but would be useful for evaluating transmission sources.

We found, that isolates belonging to GS II generally had larger genomes than isolates from GS I, and that gut mucosal and faecal isolates were more predominant in GS II. While we found candidate genes increasing and decreasing (respectively) in prevalence from saliva to gut biopsies to faeces, there were few genes that were specific to collection site. Previously, particular genes involved in sodium-hydrogen antiporting, sulfite reductase and peptidoglycan biosynthesis have been related to the pathogenic activity of *C. concisus* in IBD^9^. We found that genes involved in transport of nutrients and cell metabolism were more abundant in the enteric isolates, possibly indicating, that the intestine is a colonisation site for *C. concisus*, and relates to the metabolic activity required in this niche. Findings of no difference in the existence of *C. concisus* subtypes between clinical groups support the suggestion that *C. concisus* is not always pathogenic, and that genetic variability reflects the bacterial adaptation to different niches of the gastrointestinal tract, rather than disease status of the host. Since the pathogenic activities of *C. concisus* have been elucidated *in-vitro* studies^22,25,28^, an explanation could be that *C. concisus* is a pathobiont, which exerts pathogenic activity only when the surrounding environment is suitable, and that this characteristic is unrelated to genotype. This is consistent with evidence that there is no correlation between clinical presentation and presence of the putative virulence genes *zot* and exotoxin 9. However, we observed a relatively low prevalence of *zot* in our study (15%) compared to previous findings in which 30% of Australian oral isolates were positive^24^. Since most of the isolates in our study derive from gut mucosal biopsies, this may indicate that certain polymorphisms of the *zot* gene are only present in oral isolates from IBD patients, or that geographical differences exist. Isolates that contain both *zot* and exotoxin 9 DNA have not previously been described in detail. In this study, we found nine isolates with both virulence factors, from five different patients that all had IBD. The sample size of our present study was not powered to detect a statistically significant difference,but these results could indicate that accumulation of several virulence genes may be related to disease phenotype.

We previously found that the prevalence of *C. concisus* was considerably higher for the UC-IPAA subgroup of IBD patients compared to healthy controls^8^. Patients that have undergone UC-IPAA surgery for UC have the most severe form of disease, and we found that the majority of UC-IPAA patients in our study had continued clinical and endoscopic signs of inflammation. However, the results of our study do not indicate a correlation between genetic diversity of *C. concisus* and clinical presentation. Therefore, a possible association with disease could be related to relative quantities of *C. concisus*, instead of specific genomospecies. The etiology of IBD is multifactorial, but dysbiosis of the intestinal microbiota is believed to be a key initiating factor^29^. Given the fact that *in-vitro* studies with *C. concisus* have demonstrated pathogenic capabilities such as induction of apoptosis^30^, as well as epithelial invasion and cytokine production^25,31^, it seems plausible that pathogenic *C. concisus* isolates could be important in such dysbiotic environments. An interesting approach to understanding the *in-vivo* actions of *C. concisus* would therefore be to investigate the interactions with other enteric bacteria in health and disease. The relationship between *C. concisus* and the microbiota in five children with CD has been investigated and the prevalence of *C. concisus* was associated with increased levels of *Firmicutes* reported by abundance levels and potential genetic exchange^32,33^. Studies examining the microbial compositions of the luminal and gut mucosal flora in *C. concisus* positive patients with IBD or diarrhoea, as well as in healthy controls, would be useful for understanding the role of this enigmatic organism in intestinal inflammation.

In conclusion, molecular typing of *C. concisus* isolates from saliva, mucosa and faecal samples of IBD patients and healthy controls indicated high genetic diversity among *C. concisus* isolates regardless of clinical presentation. In general, there was a subdivision of isolates into two clusters/genomospecies, related to anatomical sampling site. We identified genetic variation associated with the population structure of *C. concisus* as well as candidate genes associated with the colonisation site in humans, notably genes involved in cell transport and metabolism, as well as cell wall/membrane biosynthesis. As our data does not support a specific disease related genotype of *C. concisus*, we suggest that the pathogenic potential may be modulated by the specific microbial environment in the gut, but further studies are needed to confirm this.

## Methods

### Isolates and patient characteristics

A total of 104 *C. concisus* isolates were sequenced and analysed in this study, sampled from 41 different adult patients. Two or more isolates were recovered from 27 patients. Of all patients, eight had ulcerative colitis (UC), three had Crohn’s disease (CD), 15 had ulcerative colitis with previous ileal-pouch-anal-anastomosis surgery (UC-IPAA), three had gastroenteritis (GE) and 12 were healthy controls (HC) (Table 3). The mean age was 49 years (range: 20–73). Fifty-four percent of participants were male (22/41). Nine isolates were from UC (1 oral, 7 biopsies, 1 faecal), 16 from CD (2 oral, 13 biopsies, 1 faecal), 41 from UC-IPAA (5 oral, 27 biopsies, 9 faecal), seven from GE (3 biopsies, 4 faecal) and 31 from HC (5 oral, 21 biopsies, 5 faecal). A detailed overview of corresponding isolates for all patients is provided in Supplementary Table 4. This includes previously sequenced strains^8^, and two faecal isolates from a study investigating isolates from diarrheic patients^3^. The majority of isolates (n = 102) derive from a previous study aimed at optimizing cultivation procedures from mucosal biopsies^8^. Isolates were randomly chosen across the sampling frame, to capture as much genetic diversity as possible. Briefly, samples for cultivation were collected from saliva, gut mucosal biopsies and faecal samples from each study participant, using the Aalborg two-step incubation procedure and cultivation using a filter technique^8,34^. From agar plates where individual and separable colonies existed, these were collected and enumerated accordingly. Isolates were stored at −80 °C until preparation for use in this study and the isolates had less than five passages on artificial media. Written informed consent was provided by all participants and the studies were approved by the Regional Ethics Committee of Northern Jutland, Denmark (N-20013070, N-20110008). All research was conducted in accordance with the Danish Health Act. In addition to our isolates, nine publically available genomes from the NCBI database were also included in the comparative analysis. These strains were sampled from gut mucosal biopsies of three patients with Crohn’s disease (UNSWCD, UNSW2, UNSW3), faecal isolates from patients with gastroenteritis (UNSW1, UNSWCS, ATCC 51562, BAA-1457 (13826)), one faecal isolate from a healthy person (ATCC 51561) and one oral isolate from a patient with periodontitis (ATCC 33237).

**Table 3:** Overview of patient characteristics by clinical group (CD: Crohn’s disease, GE: Gastroenteritis, HC: Healthy controls, UC: Ulcerative colitis, UC-IPAA: Ulcerative colitis, with previous ileal-pouch-anal-anastomosis).

Group	N	Sex, male (%)	Age, mean (range)	Number of isolates			Inflammation	
				Saliva	Mucosa	Faeces	Symptoms (%)	Endo-/microscopic (%)
CD	3	2 (33)	39 (24–63)	2	13	1	3(100)	1 (33)
UC	8	3 (37)	52 (33–68)	1	7	1	5 (62)	5 (62)
UC-IPAA	15	9 (60)	41 (21–56)	5	27	9	13 (87)	12 (80)
GE	3	1 (33)	47 (20–65)	0	3	4	3 (100)	0 (0)^*^
HC	12	7 (58)	60 (45–73)	5	21	5	—	—
Total	41	22 (44)	49 (20–73)	13	71	20	24/27 (83)	19/26 (73)

^*^Endoscopic/microscopic evaluation not performed for one patient (AAUH-2010376221).

### DNA extraction and genome sequencing

DNA was extracted using the QIAamp DNA Mini Kit (QIAGEN, Crawley, UK), according to manufacturer’s instructions. DNA was quantified using a Nanodrop spectrophotometer, as well as the Quant-iT DNA Assay Kit (Life Technologies, Paisley, UK) before sequencing. Genome sequencing was performed on an Illumina MiSeq sequencer using the Nextera XT Library Preparation Kit with standard protocols. Libraries were sequenced using 2 × 250 bp paired end v3 reagent kit (Illumina), following manufacturer’s protocols. Short read paired-end data was assembled using the de novo assembly algorithm, SPAdes (version 3.10.0^35^. The average number of contigs was 92 (range: 3–356) for an average total assembled sequence size of 1.94 Mbp (range: 1.78–2.22). The average N50 was 97693 (range: 13858–934037) and the average GC content was 38.94% (range: 37.26–39.88). An overview of assembly information is provided in Supplementary Table 5. Genomes and short data are archived on the NCBI GenBank and SRA depositories, associated with BioProject accession # PRJNA395841.

### Reference pan-genome construction

A reference pangenome was assembled from a total of 113 *C. concisus* whole genomes using a previously described method^36^. Briefly, the 104 genomes from this study and an additional 9 *C. concisus* reference genomes were automatically annotated using the SEED/RAST system^37,38^ and a pangenome reference list of unique genes was assembled using BLAST with the following parameters: a sequence was considered to be an allelic variant of an existing gene when local alignment was >70% of sequence identity on >10% of the sequence length. Any sequence below these thresholds was considered a novel gene and added to the list. The final list of genes was filtered using CD-HIT^39^ with a sequence identity cut-off of 90% nucleotide identity. A total of 4,798 unique genes were discovered and their prevalence was examined in all genomes from this study. Functional annotation of the list was made using RPSBLAST 2.2.15 program on the Clusters of Orthologous Groups (COG) database (NCBI, 28/03/2017) implemented in the WebMGA server (http://weizhong-lab.ucsd.edu/metagenomic-analysis/server/cog/). A total of 2,639/4,798 (55%) genes could be assigned to a described COG.

### Gene-by-gene analyses and genome alignments

Sequence alignments and genome content comparison analyses using BLAST were performed gene-by-gene, as implemented in the BIGSdb platform^40–42^ and described in previous *Campylobacter* studies^43–47^. Briefly, genes were scanned in genomes using BLAST with the following parameters: a gene was considered present in a given genome when its sequence aligned to a genomic sequence with >70% sequence identity on >50% of sequence length using BLAST. Genome alignments were produced^41,42^ by concatenating single-gene alignments using MAFFT^48^.

### Typing using MLST and rRNA

Multi locus sequence typing was conducted using the seven loci *aspA*, *atpA*, *glnA*, *gltA*, *glyA*, *ilvD* and *pgm*, described by Miller at al. with sequences obtained from PubMLST (http://pubmlst.org)^19^. The combination of the six loci *asd, aspA, atpA, glnA, pgi* and *tkt* previously used by Mahendran *et al*.^15^ was used for comparison, as well as typing by 16 S rRNA and 23 S rRNA sequences, all obtained from the NCBI database (https://www.ncbi.nlm.nih.gov/).

### Phylogenetic reconstruction

Phylogenetic trees were inferred using the neighbour-joining algorithm from core genome sequence alignments and visualised using MEGA7 software^49^. Data was analysed using Stata 14 (Statacorp LP, Texas, USA). The McNemar chi-squared test was used for comparison of groups, and a p-value < 0.05 was considered statistically significant.

### Data Availability

Genomes and short data are archived on the NCBI GenBank and SRA depositories, associated with 455 BioProject accession # PRJNA395841. Assembled genomes are also shared on figshare under DOI: 456 10.6084/m9.figshare.5245219. All MLST sequences are pending submission to the PubMLST 457 database for ST assignment and online accession from NCBI. (http://pubmlst.org/campylobacter/).

## Electronic supplementary material

Supplementary information
