# Supplementary material for: Molecular epidemiology and comparative genomics of Campylobacter concisus strains from saliva, faeces and gut mucosal biopsies in inflammatory bowel disease
Source: Sci Rep. 2018 Jan 30;8:1902. doi: 10.1038/s41598-018-20135-4 (PMC5790007; doi:10.1038/s41598-018-20135-4)
Supplement: Supplementary file 1 — Supplementary information [file 41598_2018_20135_MOESM1_ESM.pdf]

Molecular epidemiology and comparative genomics of *Campylobacter concisus* strains from saliva, faeces and gut mucosal biopsies in inflammatory bowel disease

Karina Frahm Kirk[1,2]*, Guillaume Méric[3], Hans Linde Nielsen[4], Ben Pascoe[3], Samuel K. Sheppard[3,5], Ole Thorlacius-Ussing[2,6], Henrik Nielsen[1,2]

[1]Department of Infectious Diseases, Aalborg University Hospital, Aalborg, Denmark. [2]Department of Clinical Medicine, Aalborg University, Aalborg, Denmark. [3]The Milner Center for Evolution, Department of Biology and Biochemistry, Bath University, Bath, United Kingdom. [4]Department of Clinical Microbiology, Aalborg University Hospital, Aalborg, Denmark. [5]Department of Zoology, University of Oxford, Oxford, UK. [6]Department of Gastrointestinal Surgery, Aalborg University Hospital, Aalborg, Denmark.

Supplementary material

*Corresponding author: Karina Frahm Kirk, kfk@rn.dk

Supplementary figure 1: A phylogenetic tree depicting the genetic relatedness of 113 *C. concisus* isolates. The tree is based on the concatenated sequences of seven *C. concisus* housekeeping genes. Each sample is annotated according to sampling site (dot=gut mucosal biopsy, triangle=saliva, diamond=faeces). Colours represent clinical presentation (red=IBD, yellow=GE, green=HC, black=reference strains). Text=BIGSdb reference and isolate ID's (more information regarding this can be found in Table S1). Reference strain *C. curvus* 525-92 is used as an outgroup.

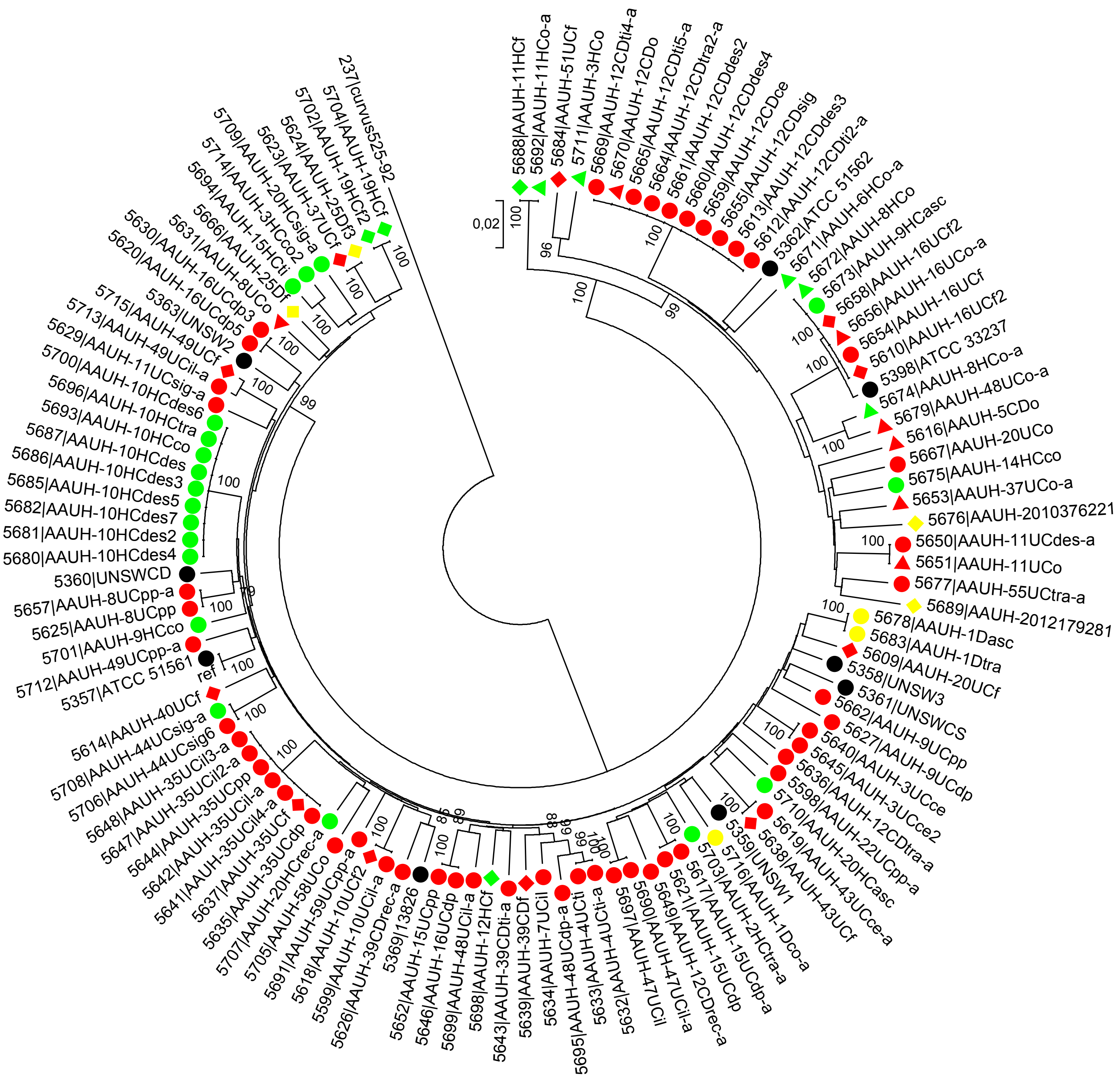
0,02
5688|AAUH-11HCf
5692|AAUH-11HCo-a
5684|AAUH-51UCf
5711|AAUH-3HCo
5669|AAUH-12CDti4-a
5670|AAUH-12CDo
5665|AAUH-12CDti5-a
5664|AAUH-12CDtra2-a
5661|AAUH-12CDdes2
5660|AAUH-12CDdes4
5659|AAUH-12CDce
5655|AAUH-12CDsig
5613|AAUH-12CDdes3
5612|AAUH-12CDti2-a
5362|ATCC 51562
5671|AAUH-6HCo-a
5672|AAUH-8HCo
5673|AAUH-9HCasc
5658|AAUH-16UCf2
5656|AAUH-16UCo-a
5654|AAUH-16UCf
5610|AAUH-16UCf2
5398|ATCC 33237
5674|AAUH-8HCo-a
5679|AAUH-48UCo-a
5616|AAUH-5CDo
5667|AAUH-20UCo
5675|AAUH-14HCco
5653|AAUH-37UCo-a
5676|AAUH-2010376221
5650|AAUH-11UCdes-a
5651|AAUH-11UCo
5677|AAUH-55UCtra-a
5689|AAUH-2012179281
5678|AAUH-1Dasc
5683|AAUH-1Dtra
5609|AAUH-20UCf
5358|UNSW3
5361|UNSWCS
5662|AAUH-9UCpp
5627|AAUH-9UCdp
5640|AAUH-3UCce
5645|AAUH-3UCce2
5636|AAUH-12CDtra-a
5598|AAUH-22UCpp-a
5710|AAUH-20HCasc
5619|AAUH-43UCce-a
5638|AAUH-43UCf
5359|UNSW1
5716|AAUH-1Dco-a
5703|AAUH-2HCtra-a
5617|AAUH-15UCdp-a
5621|AAUH-15UCdp
5649|AAUH-12CDrec-a
5690|AAUH-47UCil-a
5697|AAUH-47UCil
5632|AAUH-4UCti-a
5633|AAUH-4UCti
5695|AAUH-48UCdp-a
5634|AAUH-7UCil
5639|AAUH-39CDf
5643|AAUH-39CDti-a
5698|AAUH-12HCf
5699|AAUH-48UCil-a
5646|AAUH-16UCdp
5652|AAUH-15UCpp
5369|13826
5626|AAUH-39CDrec-a
5599|AAUH-10UCil-a
5618|AAUH-10UCf2
5691|AAUH-59UCpp-a
5705|AAUH-58UCo
5707|AAUH-20HCrec-a
5635|AAUH-35UCdp
5637|AAUH-35UCf
5641|AAUH-35UCil4-a
5642|AAUH-35UCil-a
5644|AAUH-35UCpp
5647|AAUH-35UCil2-a
5648|AAUH-35UCil3-a
5706|AAUH-44UCsig6
5708|AAUH-44UCsig-a
5614|AAUH-40UCf
5357|ATCC 51561
ref
5712|AAUH-49UCpp-a
5701|AAUH-9HCco
5625|AAUH-8UCpp
5657|AAUH-8UCpp-a
5360|UNSWCD
5680|AAUH-10HCdes4
5681|AAUH-10HCdes2
5682|AAUH-10HCdes7
5685|AAUH-10HCdes5
5686|AAUH-10HCdes3
5687|AAUH-10HCdes
5693|AAUH-10HCco
5696|AAUH-10HCtra
5700|AAUH-10HCdes6
5629|AAUH-11UCsig-a
5713|AAUH-49UCil-a
5715|AAUH-49UCf
5363|UNSW2
5620|AAUH-16UCdp5
5630|AAUH-16UCdp3
5631|AAUH-8UCo
5666|AAUH-25Df
5694|AAUH-15HCti
5714|AAUH-3HCco2
5709|AAUH-20HCsig-a
5623|AAUH-37UCf
5624|AAUH-25Df3
5702|AAUH-19HCf2
5704|AAUH-19HCf
237|curvus525-92

Supplementary table 1: Overview of genes matching COG class description according to GS.

COG class description	Number of corresponding genes in the GS I core genome	Number of corresponding genes in the GS II core genome	Difference in proportion of respective core genomes (%)
Amino acid transport and metabolism	75	127	2,323%
Carbohydrate transport and metabolism	43	82	2,130%
Cell cycle control, cell division, chromosome partitioning	13	27	0,836%
Cell motility	38	56	0,452%
Cell wall/membrane/envelope biogenesis	46	65	0,305%
Coenzyme transport and metabolism	18	27	0,255%
Defense mechanisms	12	18	0,170%
Energy production and conversion	21	30	0,167%
Function unknown	10	15	0,142%
General function prediction only	6	9	0,085%
Inorganic ion transport and metabolism	66	88	-0,020%
Intracellular trafficking, secretion, and vesicular transport	31	39	-0,212%
Lipid transport and metabolism	14	16	-0,236%
Multiple classes	76	98	-0,313%
Nucleotide transport and metabolism	44	55	-0,332%
Posttranslational modification, protein turnover, chaperones	17	18	-0,411%
Replication, recombination and repair	80	102	-0,430%
Secondary metabolites biosynthesis, transport and catabolism	24	25	-0,615%
Signal transduction mechanisms	74	84	-1,297%
Transcription	49	50	-1,347%
Translation, ribosomal structure and biogenesis	104	120	-1,653%
**Total number of genes assigned to COG**	861	1151	
**Total number of genes in respective core genomes**	975	1367	

Supplementary Table 2: Overview of genes in- and decreasing in prevalence according to sampling site

Gene name	RAST/SEED predicted function	COG predicted function	COG class	COG class description	Prevalence of gene in isolates from saliva (n=) (%)	Prevalence of gene in isolates from gastrointestinal biopsies (n=) (%)	Prevalence of gene in isolates from faeces (n=) (%)	Prevalence difference (faeces-saliva) (%)	Prevalence difference (biopsies-saliva) (%)
CCC13826_RS00275	Fur family transcriptional regulator	Fe2+/Zn2+ uptake regulation proteins	P	Inorganic ion transport and	50,0%	81,9%	87,5%	37,5%	31,9%
CCC13826_RS00390	hypothetical protein				43,8%	81,9%	83,3%	39,6%	38,2%
CCC13826_RS00400	diguanylate phosphodiesterase	FOG: EAL domain	T	Signal transduction mechanisms	12,5%	48,6%	54,2%	41,7%	36,1%
CCC13826_RS00505	CoA activase	Activator of 2-hydroxyglutaryl-CoA dehydratase (HSP70-class ATPase domain)	I	Lipid transport and metabolism	12,5%	48,6%	54,2%	41,7%	36,1%
CCC13826_RS00510	2-hydroxyglutaryl-CoA dehydratase	Benzoyl-CoA reductase/2-hydroxyglutaryl-CoA dehydratase subunit, BcrC/BadD/HgdB	E	Amino acid transport and metabolism	12,5%	47,2%	54,2%	41,7%	34,7%
CCC13826_RS00525	amidophosphoribosyltransferase	Glutamine phosphoribosylpyrophosphate amidotransferase	F	Nucleotide transport and metabolism	50,0%	84,7%	95,8%	45,8%	34,7%
CCC13826_RS00705	type VI secretion system-associated lipoprotein	Uncharacterized protein conserved in bacteria	S	Function unknown	12,5%	51,4%	54,2%	41,7%	38,9%
CCC13826_RS00715	glutamyl-tRNA amidotransferase subunit B	Uncharacterized protein conserved in bacteria	S	Function unknown	18,8%	51,4%	58,3%	39,6%	32,6%
CCC13826_RS00720	nucleobase:cation symporter				12,5%	48,6%	54,2%	41,7%	36,1%
CCC13826_RS00745	hypothetical protein	Uncharacterized protein conserved in bacteria	S	Function unknown	18,8%	52,8%	58,3%	39,6%	34,0%
CCC13826_RS01035	hypothetical protein	Hemolysin activation/secretion protein	U	Intracellular trafficking, secretion, and vesicular transport	31,3%	70,8%	75,0%	43,8%	39,6%
CCC13826_RS01495	Bcr/CflA family drug resistance efflux transporter	Na+/melibiose symporter and related transporters	G	Carbohydrate transport and metabolism	0,0%	44,4%	45,8%	45,8%	44,4%
CCC13826_RS01500	recombinase RmuC	Uncharacterized protein conserved in bacteria	S	Function unknown	12,5%	47,2%	54,2%	41,7%	34,7%
CCC13826_RS02060	pilus biosynthesis protein PilZ				43,8%	81,9%	83,3%	39,6%	38,2%
CCC13826_RS02305	malonyl CoA-acyl carrier protein transacylase	(acyl-carrier-protein) S-malonyltransferase	I	Lipid transport and metabolism	50,0%	83,3%	87,5%	37,5%	33,3%
CCC13826_RS02635	hypothetical protein				12,5%	47,2%	54,2%	41,7%	34,7%
CCC13826_RS02730	hypothetical protein				12,5%	50,0%	54,2%	41,7%	37,5%
CCC13826_RS02735	hypothetical protein	Response regulator containing CheY-like receiver, AAA-type ATPase, and DNA-binding domains	T	Signal transduction mechanisms	12,5%	47,2%	54,2%	41,7%	34,7%
CCC13826_RS03030	methyl-accepting chemotaxis protein	Methyl-accepting chemotaxis protein	NT	Multiple classes	12,5%	47,2%	62,5%	50,0%	34,7%
CCC13826_RS03455	hypothetical protein				12,5%	47,2%	54,2%	41,7%	34,7%
CCC13826_RS03605	adenylosuccinate lyase	DnaJ-class molecular chaperone with C-terminal Zn finger domain	O	Posttranslational modification, protein turnover, chaperones	12,5%	45,8%	50,0%	37,5%	33,3%
CCC13826_RS03610	hypothetical protein				12,5%	47,2%	54,2%	41,7%	34,7%
CCC13826_RS03620	tRNA pseudouridine(38-40) synthase TruA	Pseudouridylate synthase	J	Translation, ribosomal structure and biogenesis	50,0%	84,7%	91,7%	41,7%	34,7%
CCC13826_RS03720	prepilin-type N-terminal cleavage/methylation domain-containing protein	Type II secretory pathway, pseudopilin PulG	NU	Multiple classes	12,5%	48,6%	54,2%	41,7%	36,1%
CCC13826_RS03725	hypothetical protein				12,5%	48,6%	54,2%	41,7%	36,1%
CCC13826_RS04015	cell division protein FtsZ	Cell division GTPase	D	Cell cycle control, cell division, chromosome partitioning	12,5%	47,2%	54,2%	41,7%	34,7%
CCC13826_RS04045	fusaric acid resistance protein	Predicted membrane protein	S	Function unknown	31,3%	84,7%	87,5%	56,3%	53,5%
CCC13826_RS04315	membrane protein	Predicted permeases	R	General function prediction only	43,8%	84,7%	87,5%	43,8%	41,0%
CCC13826_RS04830	hypothetical protein				12,5%	47,2%	54,2%	41,7%	34,7%
CCC13826_RS04840	hypothetical protein				12,5%	47,2%	54,2%	41,7%	34,7%
CCC13826_RS04850	ATPase AAA	Uncharacterized ATPase, putative transposase	R	General function prediction only	12,5%	47,2%	54,2%	41,7%	34,7%
CCC13826_RS04855	hypothetical protein				12,5%	47,2%	54,2%	41,7%	34,7%
CCC13826_RS04860	hypothetical protein				12,5%	47,2%	54,2%	41,7%	34,7%
CCC13826_RS04880	transcriptional regulator	Methyl-accepting chemotaxis protein	NT	Multiple classes	12,5%	47,2%	54,2%	41,7%	34,7%
CCC13826_RS05745	competence protein ComEA	DNA uptake protein and related DNA-binding proteins	L	Replication, recombination and repair	12,5%	45,8%	50,0%	37,5%	33,3%
CCC13826_RS05845	lysine transporter LysE	Lysine efflux permease	R	General function prediction only	6,3%	45,8%	54,2%	47,9%	39,6%
CCC13826_RS06175	oxidoreductase	Predicted dehydrogenases and related proteins	R	General function prediction only	12,5%	48,6%	54,2%	41,7%	36,1%
CCC13826_RS06465	hypothetical protein				37,5%	75,0%	79,2%	41,7%	37,5%
CCC13826_RS06630	hypothetical protein				12,5%	44,4%	54,2%	41,7%	31,9%
CCC13826_RS06825	hypothetical protein				12,5%	47,2%	54,2%	41,7%	34,7%
CCC13826_RS06830	haloacid dehalogenase	Type II secretory pathway, pseudopilin PulG	NU	Multiple classes	12,5%	47,2%	54,2%	41,7%	34,7%
CCC13826_RS06835	prepilin-type N-terminal cleavage/methylation domain-containing protein				12,5%	44,4%	54,2%	41,7%	31,9%
CCC13826_RS06860	methyl-accepting chemotaxis protein	Methyl-accepting chemotaxis protein	NT	Multiple classes	12,5%	47,2%	54,2%	41,7%	34,7%
CCC13826_RS06885	ribose-phosphate pyrophosphokinase	Phosphoribosylpyrophosphate synthetase	FE	Multiple classes	50,0%	84,7%	95,8%	45,8%	34,7%
CCC13826_RS06960	hypothetical protein				12,5%	43,1%	54,2%	41,7%	30,6%
CCC13826_RS07065	two-component sensor histidine kinase	Signal transduction histidine kinase involved in nitrogen fixation and metabolism regulation	T	Signal transduction mechanisms	12,5%	50,0%	62,5%	50,0%	37,5%
CCC13826_RS07075	aminotransferase	Selenocysteine lyase	E	Amino acid transport and metabolism	12,5%	47,2%	54,2%	41,7%	34,7%
CCC13826_RS07080	hydroxymethylpyrimidine/phosphomethylpyrimidine kinase	Hydroxymethylpyrimidine/phosphomethylpyrimidine kinase	H	Coenzyme transport and metabolism	12,5%	47,2%	54,2%	41,7%	34,7%
CCC13826_RS07085	thiamine phosphate synthase	Thiamine monophosphate synthase	H	Coenzyme transport and metabolism	12,5%	47,2%	58,3%	45,8%	34,7%
CCC13826_RS07090	hypothetical protein				12,5%	65,3%	66,7%	54,2%	52,8%
CCC13826_RS07800	flagellar biosynthesis protein FliR	Flagellar biosynthesis pathway, component FliR	NU	Multiple classes	12,5%	50,0%	54,2%	41,7%	37,5%
CCC13826_RS08175	sufurtransferase FdhD	Uncharacterized protein required for formate dehydrogenase activity	C	Energy production and conversion	37,5%	84,7%	87,5%	50,0%	47,2%
CCC13826_RS08200	type III pantothenate kinase	Putative transcriptional regulator, homolog of Bvg accessory factor	K	Transcription	12,5%	52,8%	54,2%	41,7%	40,3%
CCC13826_RS08205	hypothetical protein				12,5%	48,6%	58,3%	45,8%	36,1%
CCC13826_RS08615	LemA family protein	Uncharacterized conserved protein	S	Function unknown	12,5%	70,8%	75,0%	62,5%	58,3%
CCC13826_RS08665	GGDEF-domain containing protein	Predicted signal transduction protein containing a membrane domain, an EAL and a GGDEF domain	T	Signal transduction mechanisms	12,5%	47,2%	54,2%	41,7%	34,7%
CCC13826_RS08730	GGDEF domain-containing protein	Predicted signal transduction protein containing a membrane domain, an EAL and a GGDEF domain	T	Signal transduction mechanisms	12,5%	47,2%	54,2%	41,7%	34,7%
CCC13826_RS08755	oxidoreductase				50,0%	84,7%	91,7%	41,7%	34,7%
CCC13826_RS08840	phosphoribosylformylglycinamidine cyclo-ligase	Predicted membrane-associated, metal-dependent hydrolase	R	General function prediction only	0,0%	31,9%	41,7%	41,7%	31,9%
CCC13826_RS09060	SAM-dependent methyltransferase	Cyclopropane fatty acid synthase and related methyltransferases	M	Cell wall/membrane/envelope biogenesis	43,8%	80,6%	83,3%	39,6%	36,8%
CCC13826_RS09330	Na+/H+ antiporter	NhaP-type Na+/H+ and K+/H+ antiporters	P	Inorganic ion transport and metabolism	43,8%	76,4%	83,3%	39,6%	32,6%
CCC13826_RS09925	hypothetical protein				43,8%	75,0%	83,3%	39,6%	31,3%
CCC13826_RS09965	hypothetical protein				12,5%	44,4%	50,0%	37,5%	31,9%
CCC13826_RS10320_pCCON31	hypothetical protein				31,3%	70,8%	75,0%	43,8%	39,6%
CCC13826_RS10480_pCCON16	hypothetical protein				43,8%	76,4%	83,3%	39,6%	32,6%
id5357_0184	Possible outer membrane protein				0,0%	37,5%	50,0%	50,0%	37,5%
id5358_0470	diguanylate cyclase (GGDEF domain) with PAS/PAC sensor	FOG: GGDEF domain	T	Signal transduction mechanisms	12,5%	47,2%	54,2%	41,7%	34,7%
id5359_1456	hypothetical protein				0,0%	33,3%	37,5%	37,5%	33,3%
id5360_0032	hypothetical protein				12,5%	48,6%	54,2%	41,7%	36,1%
id5360_1533	hypothetical protein				0,0%	38,9%	41,7%	41,7%	38,9%
id5609_0268	FIG00711288: hypothetical protein	Uncharacterized protein conserved in bacteria	S	Function unknown	0,0%	30,6%	37,5%	37,5%	30,6%
id5620_1973	hypothetical protein				6,3%	47,2%	50,0%	43,8%	41,0%
id5649_1279	Methyl-accepting chemotaxis signal transduction protein				6,3%	61,1%	70,8%	64,6%	54,9%
CCON33237_RS00065	hypothetical protein	FOG: TPR repeat, SEL1 subfamily	R	General function prediction only	75,0%	16,7%	12,5%	-62,5%	-58,3%
CCON33237_RS01715	tellurium resistance protein TerC	Membrane protein TerC, possibly involved in tellurium resistance	P	Inorganic ion transport and metabolism	87,5%	36,1%	29,2%	-58,3%	-51,4%
CCON33237_RS01690	hypothetical protein	Predicted signal transduction protein containing a membrane domain, an EAL and a GGDEF domain	T	Signal transduction mechanisms	87,5%	43,1%	33,3%	-54,2%	-44,4%
CCON33237_RS01135	hypothetical protein				87,5%	38,9%	33,3%	-54,2%	-48,6%
CCON33237_RS07640	glycosyl transferase	Glycosyltransferase involved in LPS biosynthesis	M	Cell wall/membrane/envelope biogenesis	81,3%	38,9%	33,3%	-47,9%	-42,4%
id5674_0536	UDP-glucose:(heptosyl) LPS alpha1,3-glucosyltransferase WaaG (EC 2.4.1.-)	Glycosyltransferase	M	Cell wall/membrane/envelope biogenesis	93,8%	54,2%	45,8%	-47,9%	-39,6%
CCON33237_RS08480	ligand-gated channel protein	Outer membrane receptor for monomeric catechols	P	Inorganic ion transport and metabolism	81,3%	40,3%	33,3%	-47,9%	-41,0%
CCON33237_RS07660	O-antigen ligase	Lipid A core - O-antigen ligase and related enzymes	M	Cell wall/membrane/envelope biogenesis	87,5%	43,1%	41,7%	-45,8%	-44,4%
CCON33237_RS06695	hypothetical protein				87,5%	52,8%	41,7%	-45,8%	-34,7%
CCON33237_RS00550	hypothetical protein				100,0%	55,6%	54,2%	-45,8%	-44,4%
CCON33237_RS07655	hypothetical protein	Glycosyltransferases, probably involved in cell wall biogenesis	M	Cell wall/membrane/envelope biogenesis	93,8%	56,9%	50,0%	-43,8%	-36,8%
id5362_1128	hypothetical protein				56,3%	13,9%	12,5%	-43,8%	-42,4%
CCON33237_RS07090	hydroxymethylpyrimidine/phosphomethylpyrimidine kinase	Hydroxymethylpyrimidine/phosphomethylpyrimidine kinase	H	Coenzyme transport and metabolism	87,5%	52,8%	45,8%	-41,7%	-34,7%
id5635_0653	probable uroporphyrin-III c-methyltransferase (EC 2.1.1.107)	Uncharacterized conserved protein	S	Function unknown	87,5%	51,4%	45,8%	-41,7%	-36,1%
CCON33237_RS00830	cytochrome-c peroxidase	Cytochrome c peroxidase	P	Inorganic ion transport and metabolism	87,5%	52,8%	45,8%	-41,7%	-34,7%
CCON33237_RS07110	hypothetical protein	Amidases related to nicotinamidase	Q	Secondary metabolites biosynthesis, transport and catabolism	87,5%	52,8%	45,8%	-41,7%	-34,7%
CCON33237_RS07975	GGDEF-domain containing protein	Predicted signal transduction protein containing a membrane domain, an EAL and a GGDEF domain	T	Signal transduction mechanisms	87,5%	52,8%	45,8%	-41,7%	-34,7%
id5674_1721	FIG00469601: hypothetical protein				87,5%	47,2%	45,8%	-41,7%	-40,3%
id5674_1422	DNA polymerase III beta subunit (EC 2.7.7.7)				87,5%	52,8%	45,8%	-41,7%	-34,7%
id5613_0393	hypothetical protein				50,0%	15,3%	8,3%	-41,7%	-34,7%
id5359_0677	Sensory box/GGDEF family protein	FOG: EAL domain	T	Signal transduction mechanisms	87,5%	56,9%	50,0%	-37,5%	-30,6%
CCON33237_RS04005	K+/H+ antiporter	NhaP-type Na+/H+ and K+/H+ antiporters with a unique C-terminal domain	P	Inorganic ion transport and metabolism	93,8%	62,5%	58,3%	-35,4%	-31,3%
id5616_1443	Glycosyl transferase, group 1 family protein	Glycosyltransferase	M	Cell wall/membrane/envelope biogenesis	37,5%	5,6%	4,2%	-33,3%	-31,9%
id5616_1442	UDP-glucose 4-epimerase (EC 5.1.3.2)	Nucleoside-diphosphate-sugar epimerases	MG	Multiple classes	37,5%	6,9%	4,2%	-33,3%	-30,6%

Supplementary Table 3: CRISPR elements and *C.concisus* plasmid integration genes for 141 *C. concisus* genomes, and distribution in clinical groups

	**CRISPR-associated genes**																			
	cas1_1	*cas1_2*	*cas1_3*	*cas1_4*	*cas1_5*	*cas1_6*	*cas1_7*	*cas1_8*	*cas1_9*	*cas2*	*cas2_1*	*cas2_2*	*cas3*	*cas9*	*CAS10/Csm1*	*csd*	*csd_1*	*csd_2*	*cshA*	*csm3*
Genomospecies I (n=40)	0	0	10	0	0	0	0	0	0	0	10	0	0	0	10	25	1	40	0	10
Genomospecies II (n=101)	44	1	19	1	42	1	1	1	1	82	19	1	69	1	21	60	0	34	1	21
Crohn's disease (n=28)	4	0	10	0	9	0	0	1	0	13	10	0	11	0	10	15	0	15	0	10
Gastroenteritis (n=7)	0	0	0	0	1	0	0	0	0	1	0	0	1	0	0	5	0	3	0	0
Healthy Controls (37)	13	0	9	0	11	1	0	0	0	23	9	0	19	0	10	21	1	24	0	10
Ulcerative colitis (n=19)	7	1	5	1	6	0	1	0	0	12	5	1	12	1	6	13	0	10	1	6
UC-IPAA surgery (n=40)	16	0	5	0	14	0	0	0	0	28	5	0	24	0	5	24	0	19	0	5
unknown (n=9)	4	0	0	0	1	0	0	0	1	5	0	0	2	0	0	7	0	3	0	0

	***Campylobacter concisus* plasmid integration island genes**																		
	virB2	*virB2_2*	*virB4*	*virB4_2*	*virB4_3*	*virB5*	*virB8*	*virB8_2*	*virB8_3*	*virB9*	*virB9_2*	*virB9_3*	*VirB10*	*VirB10_2*	*VirB10_3*	*VirB11*	*VirB11_2*	*VirB11_3*	*VirB11_4*
Genomospecies I (n=40)	1	4	0	1	4	1	0	1	5	0	1	5	5	0	1	7	5	0	1
Genomospecies II (n=101)	3	2	2	3	12	3	2	3	13	2	3	12	12	2	3	10	12	2	3
Crohn's disease (n=28)	3	5	0	3	8	3	0	3	9	0	3	9	9	0	3	1	9	0	3
Gastroenteritis (n=7)	1	1	0	1	0	1	0	1	1	0	1	0	0	0	1	0	0	0	1
Healthy Controls (37)	0	0	0	0	4	0	0	0	4	0	0	4	4	0	0	1	4	0	0
Ulcerative colitis (n=19)	0	0	2	0	0	0	2	0	0	2	0	0	0	2	0	4	0	2	0
UC-IPAA surgery (n=40)	0	0	0	0	4	0	0	0	4	0	0	4	4	0	0	9	4	0	0
unknown (n=9)	0	0	0	0	0	0	0	0	0	0	0	0	0	0	0	2	0	0	0

Supplementary table 4:

Overview of isolates, grouped by individual patients. The genetic relatedness of these isolates is depicted in Supplementary figure 1.

**Patient ID**	**Group**	**Age**	**Sex**	**Isolate ID**	**Source**	**Location (of biopsy)**
3UC	UC	44	M	AAUH-3UCce	Biopsy	Cecum
				AAUH-3UCce2	Biopsy	Cecum 2
4UC	UC	33	F	AAUH-4UCti	Biopsy	Terminal ileum
				AAUH-4UCti-a	Biopsy	Terminal ileum
5CD	CD	24	M	AAUH-5CDo	Saliva	
7UC	IPAA	51	F	AAUH-7UCil	Biopsy	Ileum
8UC	IPAA	41	M	AAUH-8UCo	Saliva	
				AAUH-8UCpp	Biopsy	Proximal pouch
				AAUH-8UCpp-a	Biopsy	Proximal pouch
9UC	IPAA	45	M	AAUH-9UCpp	Biopsy	Proximal pouch
				AAUH-9UCdp	Biopsy	Distal pouch
10UC	IPAA	46	M	AAUH-10UCf2	Faeces	
				AAUH-10UCil-a	Biopsy	Ileum
11UC	UC	63	F	AAUH-11UCco	Saliva	
				AAUH-11UCdes-a	Biopsy	Descending colon
				AAUH-11UCsig-a	Biopsy	Sigmoideum
12CD	CD	63	F	AAUH-12CDo	Saliva	
				AAUH-12CDti2-a	Biopsy	Terminal ileum 2
				AAUH-12CDti4-a	Biopsy	Terminal ileum 4
				AAUH-12CDti5-a	Biopsy	Terminal ileum 5
				AAUH-12CDce	Biopsy	Cecum
				AAUH-12CDtra-a	Biopsy	Transverse colon
				AAUH-12CDtra2-a	Biopsy	Transverse colon 2
				AAUH-12CDdes2	Biopsy	Descending colon 2
				AAUH-12CDdes3	Biopsy	Descending colon 3
				AAUH-12CDdes4	Biopsy	Descending colon 4
				AAUH-12CDsig	Biopsy	Sigmoideum
				AAUH-12CDrec-a	Biopsy	Rectum
15UC	IPAA	28	M	AAUH-15UCpp	Biopsy	Proximal pouch
				AAUH-15UCdp	Biopsy	Distal pouch
				AAUH-15UCdp-a	Biopsy	Distal pouch
16UC	IPAA	48	F	AAUH-16UCo-a	Saliva	
				AAUH-16UCdp	Biopsy	Distal pouch
				AAUH-16UCdp3	Biopsy	Distal pouch 3
				AAUH-16UCdp5	Biopsy	Distal pouch 5
				AAUH-16UCf	Faeces	
				AAUH-16UCf2	Faeces	
				AAUH-16UCf3	Faeces	
20UC	IPAA	56	F	AAUH-20UCco	Saliva	
				AAUH-20UCf	Faeces	
22UC	IPAA	44	M	AAUH-22UCpp-a	Biopsy	Proximal pouch
35UC	IPAA	23	F	AAUH-35UCil-a	Biopsy	Ileum
				AAUH-35UCil2-a	Biopsy	Ileum 2
				AAUH-35UCil3-a	Biopsy	Ileum 3
				AAUH-35UCil4-a	Biopsy	Ileum 4

				AAUH-35UCpp	Biopsy	Proximal pouch
				AAUH-35UCdp	Biopsy	Distal pouch
				AAUH-35UCf	Faeces	
37UC	IPAA	49	M	AAUH-37UCo-a	Saliva	
				AAUH-37UCf	Faeces	
39CD	CD	30	M	AAUH-39CDti-a	Biopsy	Terminal ileuum
				AAUH-39CDrec-a	Biopsy	Rectum
				AAUH-39CDf	Faeces	
40UC	UC	51	M	AAUH-40UCf	Faeces	
43UC	UC	67	F	AAUH-43UCce-a	Biopsy	Cecum
				AAUH-43UCf	Faeces	
44UC	UC	36	M	AAUH-44UCsig-a	Biopsy	Sigmoideum
				AAUH-44UCsig6	Biopsy	Sigmoideum 6
47UC	IPAA	38	F	AAUH-47UCil	Biopsy	Ileum
				AAUH-47UCil-a	Biopsy	Ileum
48UC	IPAA	21	M	AAUH-48UCo-a	Saliva	
				AAUH-48UCil-a	Biopsy	Ileum
				AAUH-48UCdp-a	Biopsy	Distal pouch
49UC	IPAA	38	F	AAUH-49UCil-a	Biopsy	Ileum
				AAUH-49UCpp-a	Biopsy	Proximal pouch
				AAUH-49UCf	Faeces	Faeces
51UC	IPAA	41	M	AAUH-51UCf	Faeces	
55UC	UC	57	F	AAUH-55UCtra-a	Biopsy	Transverse colon
58UC	UC	68	F	AAUH-58UCo	Saliva	
59UC	IPAA	42	M	AAUH-59UCpp-a	Biopsy	Proximal pouch
2HC	HC	73	M	AAUH-2HCtra	Biopsy	Transverse colon
3HC	HC	44	M	AAUH-3HCo	Saliva	
				AAUH-3HCce2	Biopsy	Cecum 2
6HC	HC	66	M	AAUH-6HCo-a	Saliva	
8HC	HC	45	F	AAUH-8HCo	Saliva	
				AAUH-8HCo-a	Saliva	
9HC	HC	57	M	AAUH-9HCce	Biopsy	Cecum
				AAUH-9HCasc	Biopsy	Ascending colon
10HC	HC	63	F	AAUH-10HCce	Biopsy	Cecum
				AAUH-10HCdes	Biopsy	Descending colon
				AAUH-10HCdes2	Biopsy	Descending colon 2
				AAUH-10HCdes3	Biopsy	Descending colon 3
				AAUH-10HCdes4	Biopsy	Descending colon 4
				AAUH-10HCdes5	Biopsy	Descending colon 5
				AAUH-10HCdes6	Biopsy	Descending colon 6
				AAUH-10HCdes7	Biopsy	Descending colon 7
				AAUH-10HCtra	Biopsy	Transverse colon
11HC	HC	66	F	AAUH-11HCf	Faeces	
				AAUH-11HCo-a	Saliva	
12HC	HC	56	M	AAUH-12HCf	Faeces	
14HC	HC	69	M	AAUH-14HCce	Biopsy	Cecum
15HC	HC	53	M	AAUH-15HCti	Biopsy	Terminal ileum
19HC	HC	67	F	AAUH-19HCf	Faeces	
				AAUH-19HCf2	Faeces	
20HC	HC	49	F	AAUH-20HCasc	Biopsy	
				AAUH-20HCsig-a	Biopsy	Sigmoideum

				AAUH-20HCrec-a	Biopsy	Rectum
1D	D	57	F	AAUH-1Dce	Biopsy	Cecum
				AAUH-1Dasc	Biopsy	Ascending colon
				AAUH-1Dtra	Biopsy	Transverse colon
				AAUH-2012179281	Faeces	*
2010376221	D	65	M	AAUH-2010376221	Faeces	
2D	D	20	F	AAUH-25Df	Faeces	
				AAUH-25Df3	Faeces	**
Ref.	HC			ATCC 51561	Faeces	
Ref.	CD	12	M	UNSW3	Biopsy	
Ref.	D			UNSW1	Faeces	
Ref.	CD			UNSWCD	Biopsy	
Ref.	D			UNSWCS	Faeces	
Ref.	D			ATCC 51562	Faeces	
Ref.	CD	3	M	UNSW2	Biopsy	
Ref.	D			13826	Faeces	
Ref.				ATCC 33237	Saliva	

Abbreviations: CD: Crohn's disease, UC: Ulcerative colitis, IPAA: J-pouch, HC: healthy control, D:Diarrhea. Isolate ID's indicate patient and isolate source. Isolates ending in –a indicate derivation from cultivation in anaerobic atmosphere, all others derive from microaerobic incubation. Ref.: reference isolates from ncbi.gov. Individual isolates from the same location (i.e. same agarplate) are numerated accordingly. Isolates with only numerical IDs derive from previous studies on patients with persistent diarrhea.* Isolate from faeces taken 4 years prior to isolates from the current study **Isolate derived from different faecal sample taken three months after initial faecal sample and after two weeks treatment with ciprofloxacin.

Supplementary table 5:

Overview of genome assembly information

	Isolate ID	Disease	Source	Genome size (Mbp)	Contigs	N50	GC content (%)
1	AAUH-3UCce	UC	Biopsy	1.93	44	103045	39.49
2	AAUH-3UCce2	UC	Biopsy	1.93	38	101107	39.50
3	AAUH-4UCti	UC	Biopsy	1.98	54	97028	39.26
4	AAUH-4UCti-a	UC	Biopsy	1.98	51	97971	39.20
5	AAUH-5CDo	CD	Saliva	2.10	183	89584	37.26
6	AAUH-7UCil	IPAA	Biopsy	1.98	58	95686	39.44
7	AAUH-8UCo	IPAA	Saliva	1.99	41	154956	39.48
8	AAUH-8UCpp	IPAA	Biopsy	2.02	3	82883	39.46
9	AAUH-8UCpp-a	IPAA	Biopsy	1.87	92	39071	39.66
10	AAUH-9UCpp	IPAA	Biopsy	1.82	47	79679	39.64
11	AAUH-9UCdp	IPAA	Biopsy	2.00	102	59846	39.46
12	AAUH-10UCf2	IPAA	Faeces	1.99	36	180736	39.59
13	AAUH-10UCil-a	IPAA	Biopsy	2.08	103	180889	39.41
14	AAUH-11UCo	UC	Saliva	1.89	112	161715	37.46
15	AAUH-11UCdes-a	UC	Biopsy	1.91	51	58626	37.30
16	AAUH-11UCsig-a	UC	Biopsy	1.99	31	159029	39.48
17	AAUH-12CDo	CD	Saliva	1.81	29	197548	37.56
18	AAUH-12CDti2-a	CD	Biopsy	1.81	9	934037	37.56
19	AAUH-12CDti4-a	CD	Biopsy	1.81	34	83693	37.57
20	AAUH-12CDti5-a	CD	Biopsy	1.82	29	187882	37.59
21	AAUH-12CDce	CD	Biopsy	1.86	31	483873	37.46
22	AAUH-12CDtra-a	CD	Biopsy	1.97	58	63299	39.42
23	AAUH-12CDtra2-a	CD	Biopsy	1.81	24	266692	37.59
24	AAUH-12CDdes2	CD	Biopsy	1.85	49	158794	37.53
25	AAUH-12CDdes3	CD	Biopsy	1.81	54	77176	37.64
26	AAUH-12CDdes4	CD	Biopsy	1.85	26	158715	37.46
27	AAUH-12CDsig	CD	Biopsy	1.90	20	257808	37.34
28	AAUH-12CDrec-a	CD	Biopsy	1.91	52	102065	39.63
29	AAUH-15UCpp	IPAA	Biopsy	1.90	36	162832	39.66
30	AAUH-15UCdp	IPAA	Biopsy	2.06	57	52907	39.47
31	AAUH-15UCdp-a	IPAA	Biopsy	2.03	130	35098	39.51
32	AAUH-16UCo-a	IPAA	Saliva	1.88	43	151248	37.68
33	AAUH-16UCdp	IPAA	Biopsy	1.92	57	162832	39.69
34	AAUH-16UCdp3	IPAA	Biopsy	1.99	66	64884	39.67
35	AAUH-16UCdp5	IPAA	Biopsy	2.04	39	142542	39.48
36	AAUH-16UCf	IPAA	Faeces	1.89	33	392237	37.68
37	AAUH-16UCf2	IPAA	Faeces	1.88	61	64374	37.67
38	AAUH-16UCf3	IPAA	Faeces	1.99	66	64884	39.67
39	AAUH-20UCo	IPAA	Saliva	1.81	24	235464	37.58
40	AAUH-20UCf	IPAA	Faeces	1.91	81	45189	39.70
41	AAUH-22UCpp-a	IPAA	Biopsy	2.02	44	131008	39.58
42	AAUH-35UCil-a	IPAA	Biopsy	1.93	55	97085	39.54
43	AAUH-35UCil2-a	IPAA	Biopsy	1.93	41	138370	39.52
44	AAUH-35UCil3-a	IPAA	Biopsy	1.93	39	97130	39.52
45	AAUH-35UCil4-a	IPAA	Biopsy	1.94	61	80285	39.49
46	AAUH-35UCpp	IPAA	Biopsy	1.93	40	134251	39.47
47	AAUH-35UCdp	IPAA	Biopsy	1.96	70	138943	39.55
48	AAUH-35UCf	IPAA	Faeces	1.97	76	87016	39.52
49	AAUH-37UCo-a	IPAA	Saliva	1.90	41	89957	37.49
50	AAUH-37UCf	IPAA	Faeces	2.00	54	87543	39.52

51	AAUH-39CDti-a	CD	Biopsy	1.93	62	120353	39.47
52	AAUH-39CDrec-a	UCD	Biopsy	2.01	94	42323	39.38
53	AAUH-39CDf	CD	Faeces	1.96	54	136855	39.51
54	AAUH-40UCf	UC	Faeces	2.11	178	34887	39.42
55	AAUH-43UCce-a	UC	Biopsy	2.01	118	73289	39.76
56	AAUH-43UCf	UC	Faeces	1.97	67	62023	39.64
57	AAUH-44UCsig-a	UC	Biopsy	2.05	111	54029	39.40
58	AAUH-44UCsig6	UC	Biopsy	2.02	235	16737	39.56
59	AAUH-47UCil	IPAA	Biopsy	1.94	69	70435	39.78
60	AAUH-47UCil-a	IPAA	Biopsy	1.92	90	42174	39.81
61	AAUH-48UCo-a	IPAA	Saliva	1.88	80	45066	37.58
62	AAUH-48UCil-a	IPAA	Biopsy	1.97	58	65623	39.70
63	AAUH-48UCdp-a	IPAA	Biopsy	1.94	202	19218	39.78
64	AAUH-49UCil-a	IPAA	Biopsy	2.12	160	43381	39.27
65	AAUH-49UCpp-a	IPAA	Biopsy	2.1	162	52807	39.59
66	AAUH-49UCf	IPAA	Faeces	2.22	118	97148	39.15
67	AAUH-51UCf	IPAA	Faeces	1.9	61	100202	37.29
68	AAUH-55UCtra-a	UC	Biopsy	1.88	88	37875	37.43
69	AAUH-58UCo	UC	Saliva	2.02	105	34563	39.47
70	AAUH-59UCpp-a	IPAA	Biopsy	1.92	49	102305	39.62
71	AAUH-2HCtra	HC	Biopsy	1.99	84	52482	39.42
72	AAUH-3HCo	HC	Saliva	2.03	336	26870	37.93
73	AAUH-3HCce2	HC	Biopsy	2.07	328	33756	39.88
74	AAUH-6HCo-a	HC	Saliva	1.78	85	32468	37.59
75	AAUH-8HCo	HC	Saliva	1.78	146	23267	37.76
76	AAUH-8HCo-a	HC	Saliva	1.81	132	25221	37.64
77	AAUH-9HCce	HC	Biopsy	1.98	189	21463	39.71
78	AAUH-9HCasc	HC	Biopsy	1.80	86	41150	37.68
79	AAUH-10HCce	HC	Biopsy	1.94	84	43541	39.61
80	AAUH-10HCdes	HC	Biopsy	1.91	55	65110	39.58
81	AAUH-10HCdes2	HC	Biopsy	1.90	79	59463	39.62
82	AAUH-10HCdes3	HC	Biopsy	1.91	59	74769	39.59
83	AAUH-10HCdes4	HC	Biopsy	1.90	90	45360	39.64
84	AAUH-10HCdes5	HC	Biopsy	1.91	84	39333	39.60
85	AAUH-10HCdes6	HC	Biopsy	1.98	102	36601	39.63
86	AAUH-10HCdes7	HC	Biopsy	1.90	74	50724	39.62
87	AAUH-10HCtra	HC	Biopsy	1.95	72	60348	39.59
88	AAUH-11HCf	HC	Faeces	1.92	229	14154	37.71
89	AAUH-11HCo-a	HC	Saliva	1.93	218	14905	37.70
90	AAUH-12HCf	HC	Faeces	1.96	161	22427	39.49
91	AAUH-14HCce	HC	Biopsy	1.83	157	21790	37.66
92	AAUH-15HCti	HC	Biopsy	1.95	129	24790	39.84
93	AAUH-19HCf	HC	Faeces	2.01	85	48338	39.51
94	AAUH-19HCf2	HC	Faeces	1.98	155	22062	39.64
95	AAUH-20HCasc	HC	Biopsy	2.04	319	20031	39.81
96	AAUH-20HCsig-a	HC	Biopsy	2.04	152	33006	39.43
97	AAUH-20HCrec-a	HC	Biopsy	2.00	82	84768	39.31
98	AAUH-1Dce	D	Biopsy	2.2	356	48282	39.56
99	AAUH-1Dasc	D	Biopsy	1.88	234	13858	39.77
100	AAUH-1Dtra	D	Biopsy	1.91	93	39458	39.56
101	AAUH-2012179281	D	Faeces	1.92	34	138913	37.35
102	AAUH-2010376221	D	Faeces	1.85	84	48012	37.67
103	AAUH-25Df	D	Faeces	1.81	31	134513	39.74
104	AAUH-25Df3	D	Faeces	2.01	51	121167	39.38
	ATCC 51561						

	UNSW3 (ref)						
	UNSW1 (ref)						
	UNSWCD (ref)						
	UNSWCS (ref)						
	ATCC 51562 (ref)						
	UNSW2 (ref)						
	13826 (BAA-1457) (ref)						
	ATCC 33237 (ref)						

Table 2: Genome assembly information and NCBI accession numbers. CD: Crohn’s disease, UC: Ulcerative colitis, IPAA: Ileal-pouch-anal-anastomosis (J-pouch), HC: healthy control
